# Multiplex array proteomics detects increased MMP-8 in CSF after spinal cord injury

**DOI:** 10.1186/1742-2094-9-122

**Published:** 2012-06-11

**Authors:** Matthew Light, Kenneth H Minor, Peter DeWitt, Kyle H Jasper, Stephen JA Davies

**Affiliations:** 1 Department of Neurosurgery, University of Colorado School of Medicine, Building RC-1 North, Room P18-9400, 12800 E 19th Avenue, Aurora, CO, 80045, USA; 2 Colorado Biostatistics Consortium, Department of Biostatistics and Informatics, University of Colorado Denver, 12477 E 19th Avenue, Room 102, Aurora, CO, 80045, USA

**Keywords:** biomarker, cerebrospinal fluid (CSF), cytokine, inflammation, matrix metalloproteinase-8 (MMP-8), microarray, proteomics, spinal cord injury, tissue inhibitor of metalloproteinase-1 (TIMP-1).

## Abstract

****Introduction**:**

A variety of methods have been used to study inflammatory changes in the acutely injured spinal cord. Recently novel multiplex assays have been used in an attempt to overcome limitations in numbers of available targets studied in a single experiment. Other technical challenges in developing pre-clinical rodent models to investigate biomarkers in cerebrospinal fluid (CSF) include relatively small volumes of sample and low concentrations of target proteins. The primary objective of this study was to characterize the inflammatory profile present in CSF at a subacute time point in a clinically relevant rodent model of traumatic spinal cord injury (SCI). Our other aim was to test a microarray proteomics platform specifically for this application.

****Methods**:**

A 34 cytokine sandwich ELISA microarray was used to study inflammatory changes in CSF samples taken 12 days post-cervical SCI in adult rats. The difference between the median foreground signal and the median background signal was measured. Bonferroni and Benjamini-Hochburg multiple testing corrections were applied to limit the False Discovery Rate (FDR), and a linear mixed model was used to account for repeated measures in the array.

****Results**:**

We report a novel subacute SCI biomarker, elevated levels of matrix metalloproteinase-8 protein in CSF, and discuss application of statistical models designed for multiplex testing.

****Conclusions**:**

Major advantages of this assay over conventional methods include high-throughput format, good sensitivity, and reduced sample consumption. This method can be useful for creating comprehensive inflammatory profiles, and biomarkers can be used in the clinic to assess injury severity and to objectively grade response to therapy.

## **Introduction**

Traumatic injury to the spinal cord can result in life-changing neurological deficits. Patients, family members, clinicians, and researchers alike face significant long-term challenges including (but not limited to) pain management, rehabilitation, and functional improvement. According to 2011 estimates, the incidence of spinal cord injury (SCI) is roughly 12,000 cases per year in the USA, 40% resulting from motor vehicle accidents and 27% result from falls, creating a bimodal age distribution in the patient population [1]. Long-term implications of these debilitating injuries are significant. Advances in patient care, specifically prevention and treatment of urinary tract complications and renal failure, have improved longevity such that causes of mortality are now similar to the general population (cardiovascular disease, cancer, and lower respiratory disease) [2,3].

The molecular mechanisms underlying inflammation-induced damage and recovery of function at the acute, subacute and chronic stages after SCI are currently being pieced together. Although trauma to the spinal cord results in rapid loss of neurons and glia within gray and white matter and transection of white matter pathways, this acute insult also initiates a robust inflammatory response [4-6] that continues to damage tissue [7,8], creating new deficits at subacute and chronic time points post-injury. Loss of blood-spinal cord barrier (BSCB) [9] and lymphocytic infiltrate into the injury site [10] potentiates inflammation, and reactive gliosis and formation of scar tissue prevent axon regeneration [5,11,12].

With clinical trials testing the effects of new therapies on neurologic outcomes post-SCI already underway (minocycline and erythropoetin to name a few) [13], and more still in the scientific pipeline, the role of cerebrospinal fluid (CSF) biomarkers as indicators of injury progression and repair is now more important than ever. Although functional improvement remains the gold standard and certainly the most important outcome for patients, Kwon *et al.* highlight the potential role for biomarkers in assessing initial injury severity and tracking changes in injury status over time. In addition, they emphasize the role of biomarkers in determining therapeutic response more objectively than current highly subjective functional outcome measures like the American Spinal Injury Association (ASIA) Score [14]. Other important potential applications include characterization of molecular signs of neurotoxic damage and endogenous neuro-repair, and discovery of new therapeutic targets. What is needed is a protein-based high-throughput assay that can detect biomarkers from a safe and clinically available source. Sensitivity is also critical because CSF (an important source of biomarkers given its proximity to neuronal tissue) contains relatively low protein concentrations even after BSCB disruption [15]. This ideal assay would be of significant value to researchers and clinicians alike, facilitating delivery of new therapies to SCI patients.

It is becoming increasingly apparent that inflammation plays key roles in both acute and secondary damage, and late-phase recovery after traumatic SCI, and that any novel therapy would almost certainly interact with these processes. In fact, much can be learned from the Immunology field, where researchers have adopted many of the latest technologies to characterize inflammatory processes previously too complex for traditional methods of investigation. Where PCR and standard ELISA were once popular for detecting changes in mRNA and protein respectively, newer multiplex arrays have emerged as the current assays of choice. Recently Lee *et al.* simultaneously profiled 10 different cytokine responses to H1N1 infection in hospitalized patients using a cytometric bead array system [16], and Mizutani *et al.* were able to track serial changes in 17 cytokines using a similar suspension array in patients with Crohn’s disease treated with Infliximab [17]. Oncology is another field that has made good on the promise of newer array technology. Tefferi *et al.* studied the prognostic value of 30 cytokines in primary myelofibrosis by multiplex sandwich immunoassay [18]: one of many truly comprehensive cytokine profiling studies. For a thorough review of multiplex sandwich ELISA technology read Nielsen and Geierstanger [19].

An effort has also been made to validate the reliability of these new assays, focusing mainly on the issues surrounding transition from a uniplex to multiplex format. Pang *et al.* studied two commercially available solution-phase microarray platforms against standard ELISA in detecting autoantibodies in human serum [20]. They note similar detection performance, and name high-throughput screening and reduced reagent consumption as major advantages over standard methods. This paper also emphasizes the importance of cutoff values (criteria for determining positive and negative signals in the dataset). In further considering data processing, the multiplex format necessitates different statistical treatment of the data: the need for multiple hypothesis testing. The concept of the false discovery rate (FDR) comes from earlier work in epidemiology and genome-wide association studies: the idea that as the number of statistical tests performed increases, so does the number of false positives. Hsueh *et al.* compare strategies like Benjamini-Hochburg [21] for estimating the FDR, and emphasize the importance of controlling the type I error rate in multiplicity testing [22]. Finally, important technical issues such as binding ‘inhomogeneity’ across immobilized spot-based arrays [23] are currently under investigation. To summarize, multiplex proteomic assays, although relatively new, offer many qualities of the ‘ideal assay’ described above, provided there is: correct statistical treatment of the data, careful consideration of technical issues, and verification of ‘hits’ with appropriate confirmatory methods.

The challenge of characterizing the inflammatory response to acute SCI has been met using a variety of methods from flow cytometry, to PCR, to ELISA. Streit *et al.* showed transient increases in IL-1β, TNF-α, IL-6, and M-CSF mRNA by PCR from cord tissue in a rat model of SCI (C8 contusion) [24]. Wang *et al.* also showed elevated IL-1β mRNA in contused cord, but extended their analysis by showing elevated protein levels in cord by ELISA. Of note, CSF samples also contained low levels of IL-1β [25]. These studies, and others, established the presence of inflammatory molecules in the setting of acute SCI, and yet they were clearly limited by the number of molecules that could be studied. More recently, the emergence of high-throughput multiplex techniques has allowed for more comprehensive analyses. Kwon *et al.* studied 25 cytokines (as well as a number of growth factors) simultaneously in human CSF samples from acute SCI patients [26]. Lubieniecka *et al.* identified 42 possible biomarkers (cytokines, growth factors, etc.) in CSF from a rat model 24 hours post-injury by mass spectrometry of which 10 correlated with injury severity (ASIA Score) including Ywhaz, Itih4, and Gpx3 [27].

In an effort to characterize inflammatory markers present at subacute time points after traumatic SCI, thought to represent a clinically relevant ‘window of opportunity’ for therapeutic intervention, we have conducted a proteomics analysis of CSF from rats at 12 days after cervical spinal cord contusion for changes in 34 cytokines using a multiplex sandwich ELISA microarray. Initial statistical analysis yielded seven proteins (IL-1α, Leptin, B7-2/CD86, GM-CSF, IL-1β, matrix metalloproteinase-8 (MMP-8), and tissue inhibitor of metalloproteinase-1 (TIMP-1)), the levels of which were all increased in the CSF from injured animals compared with normal uninjured controls. The FDR was controlled (limit type I errors) by adjusting the *P*-values using both Benjamini-Hochburg and Bonferroni methods (although we chose to focus our analysis on Benjamini-Hochburg adjustments because this method generally allows more potential hits to reach significance). To account for technical replicates, as well as variation in regional signal intensity within the array, a linear mixed model was applied, which showed only MMP-8 was significantly elevated in injured CSF, a finding subsequently confirmed by western blot.

## **Methods**

### **Spinal cord injury model**

Animal care and experimental protocols were carried out according to the guidelines of the National Institutes of Health as well as the policies of our Institutional Animal Care and Use Committee at the University of Colorado Anschutz Medical Campus. Female Sprague–Dawley rats (Harlan Laboratories, Denver, CO, USA) aged between 3 months and 5 months were used. Animals were anesthetized with an intraperitoneal injection of ketamine and xylazine, shaved and placed in a stereotactic device for small rodent surgery (David Kopf Instruments, Tujunga, CA, USA). Lubricating eye gel was applied to prevent drying under anesthesia. After cleaning the surgical site, a midline incision was made extending from the shoulder blades to the base of the skull. Another midline incision at the origin of the trapezius muscles was made, and the paraspinal muscles were scraped from the spinous processes and lamina of C2 to C6 with a small scalpel blade, exposing the spinal column. Hemostasis was achieved using gauze with light pressure. Once the spinal column was exposed, the posterior longitudinal ligament was cut above and below C4. Rongeurs were used to perform a C4 laminectomy, keeping the dura intact.

An Infinite Horizon impactor (Precision Systems and Instrumentation, Fairfax, VA, USA) with a 1.7 mm tip was used to create a unilateral dorsolateral funiculus contusion of the cervical cord. After laminectomy the animal was secured into the impactor by clamping the vertebral bodies of C3 and C5 using Adson stabilizing forceps. Contusion was made by dropping a weight with a preset force of 75 kdynes on the lateral aspect of the cord at the C4 level with a dwell time set at zero. Both displacement and force were recorded to minimize injury variation. The animal was then returned to the stereotactic device. The trapezius muscles were approximated using simple interrupted stitches (4–0 size vicryl suture). Skin was closed with surgical staples.

### **Cerebrospinal fluid collection**

**Figure 1:**
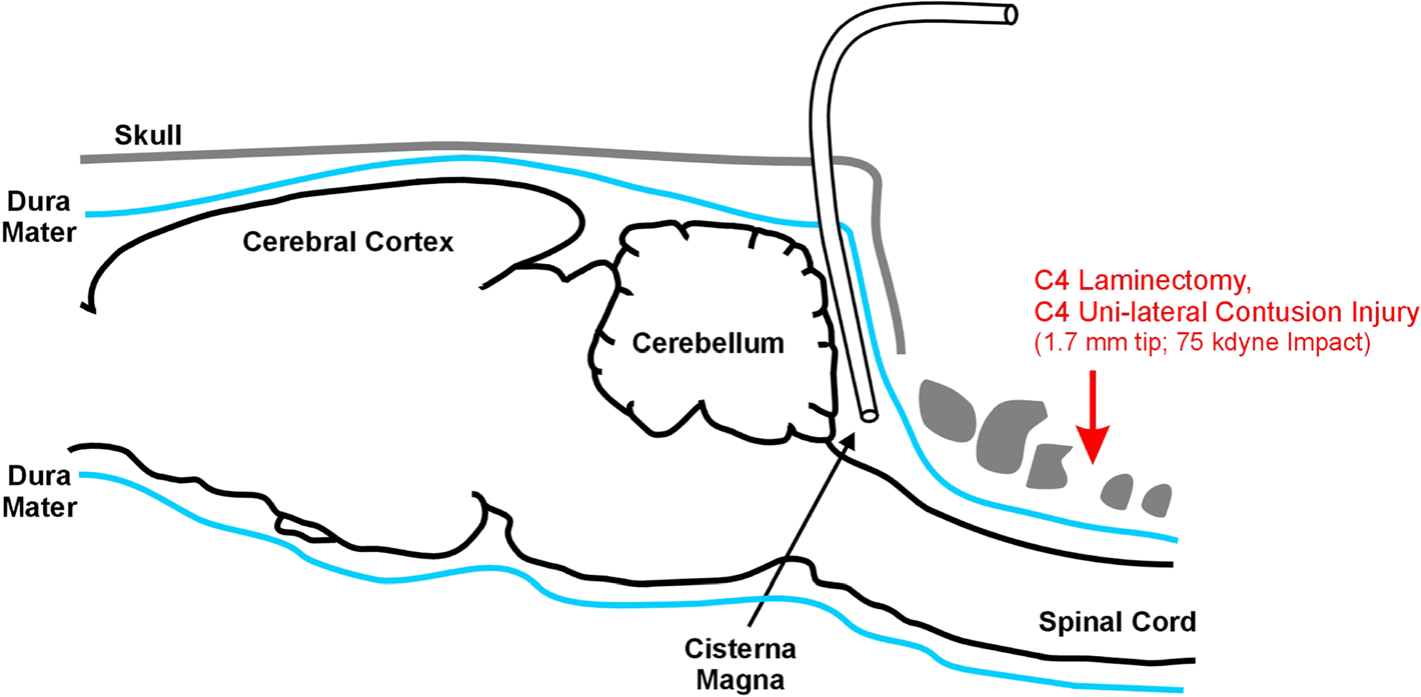
**Schematic of contusion injury and cannula placement for CSF extraction.** The cannula was guided to a depth of 7 mm to access the cisterna magna below the cerebellum.

Animals were again anesthetized at 12 days following contusion and were placed in the stereotactic device. The old incision was extended rostrally between the ears to expose the occipital bone of the skull. Subcutaneous tissue was scraped off the scull with a scalpel, and a small burr hole (from 1 mm to 2 mm diameter) was drilled through the occipital bone, exposing the meninges. A slit was then made in the dura and a small diameter cannula (order number 7741, Durect Corp., Cupertino, CA, USA) was carefully guided under the dura (in a caudal direction) along the internal surface of the occipital bone and into the cisterna magna (depth of 7 mm). A syringe was used to draw approximately 200 μL of CSF from each animal, making sure the sample was not bloody. All grossly bloody taps were excluded from the analysis (three in total). Protease inhibitor cocktail (Sigma-Aldrich, St. Louis, MO, USA) was quickly added and samples were placed on dry ice. Samples were stored at −80°C until further use. Animals were then sacrificed by intrahepatic injection of ketamine and xylazine. Control CSF was obtained from age-matched animals (no contusion) in a similar fashion. Figure 1 shows a schematic of injury location and cannula placement for CSF extraction.

### **Cerebrospinal fluid analysis**

**Figure 2:**
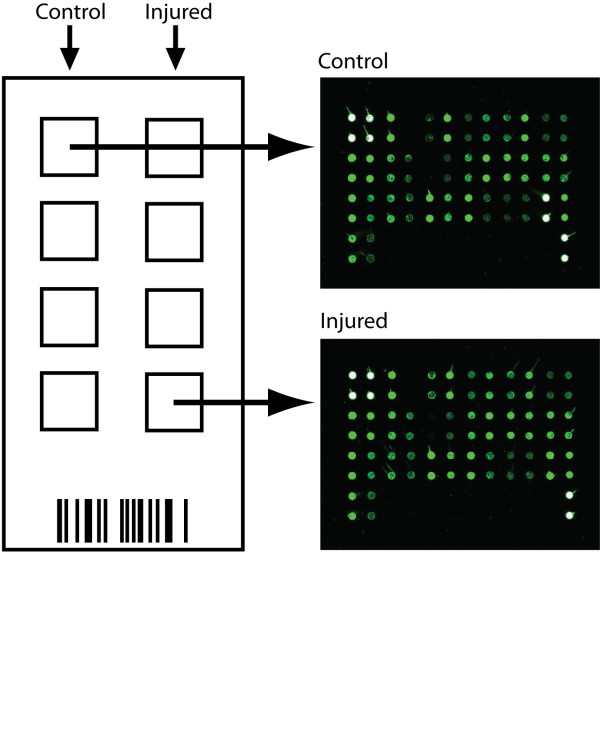
**Array format. (Left)** Schematic of microarray chip layout. Control and injured CSF were plated in adjacent columns, *n* = 4 for each condition. **(Right)** Image of representative control **(Top)** and injured **(Bottom)** CSF arrays in the Cy3 channel. A key to the location of spotted primary antibodies is shown in Table1.

Samples were removed from −80°C, thawed on ice, and briefly centrifuged. CSF from injured animals was compared with control CSF (*n* = 4) using the 34 cytokine preconfigured sandwich ELISA Rat Cytokine Array G2 (RayBiotech, Norcross, GA, USA). To reduce batch variability100 μL of each CSF sample (eight total) occupied a single array such that only one chip was used for the experiment. CSF samples were not diluted. The chip was read using a GenePix 4000B Microarray Scanner (Molecular Devices, Sunnyvale, CA, USA). Of note, the array was arranged such that each antibody was spotted twice, creating two technical replicates per protein of interest. Any remaining CSF was aliquoted and placed back at −80°C. Specific protocol details can be found at the website of RayBiotech Inc [28]. (http://www.raybiotech.com). A schematic of the chip format and image of representative control and injured arrays in the Cy3 channel are shown in Figure 2. Layout of spotted primary antibodies is shown in Table 1 (adapted from RayBiotech Inc.).

**Table 1:** Primary antibody key

**Pos**	**Pos**	**Pos**	**Neg**	**Act A**	**Agrin**	**B7-2/CD86**	**β-NGF**	**CINC-1**	**CINC-2α**	**CINC-3**	**CNTF**
**Pos**	**Pos**	**Pos**	**Neg**	**Act A**	**Agrin**	**B7-2/CD86**	**β-NGF**	**CINC-1**	**CINC-2α**	**CINC-3**	**CNTF**
**Fas-L**	**Fract**	**GM-CSF**	**ICAM-1**	**IFN-γ**	**IL-1α**	**IL-1β**	**IL-1 R6**	**IL-2**	**IL-4**	**IL-6**	**IL-10**
**Fas-L**	**Fract**	**GM-CSF**	**ICAM-1**	**IFN-γ**	**IL-1α**	**IL-1β**	**IL-1 R6**	**IL-2**	**IL-4**	**IL-6**	**IL-10**
**IL-13**	**Leptin**	**LIX**	**L-Sel**	**MCP-1**	**MIP-3α**	**MMP-8**	**PDGF-AA**	**PRL- R**	**RAGE**	**TK-1**	**TIMP-1**
**IL-13**	**Leptin**	**LIX**	**L-Sel**	**MCP-1**	**MIP-3α**	**MMP-8**	**PDGF-AA**	**PRL-R**	**RAGE**	**TK-1**	**TIMP-1**
**TNF-α**	**VEGF**	**Neg**	**Neg**	**Neg**	**Neg**	**Neg**	**Neg**	**Neg**	**Neg**	**Neg**	**Neg**
**TNF-α**	**VEGF**	**Neg**	**Neg**	**Neg**	**Neg**	**Neg**	**Neg**	**Neg**	**Neg**	**Neg**	**Pos**

Antibody layout for each array on microarray chip AAR-CYT-G2 (image adapted from RayBiotech). Each primary antibody is spotted twice per array; eight arrays are spotted per chip. Positive and negative controls determine signal variation between arrays.

### **Statistical analysis**

Analysis of the data was by the following steps. The dataset was read into R [29] and each of the 96 probe sets (includes cytokines, positive, and negative controls) were tested for significance. A two sample, equal variance *t*-test was used to test the hypothesis H0: μ1 = μ2 versus H1: μ1 ≠ μ2 where μ1 is the mean response for the injured rats and μ2 is the mean response for the non-injured rats. The response in this case is the difference between the median foreground signal and the median background signal on the Cy3 channel. Raw *P*-values for each of the 96 probe sets were recorded. Bonferroni and Benjamini-Hochburg multiple testing corrections were used to adjust *P*-values. Accounting for the technical replicates was done using linear mixed models and done in R via the nlme package [30].

### **Western blot analysis**

#### ** *MMP-8 western blot* **

The same CSF samples that had been used for microarray were removed from −80°C and thawed on ice. Samples (30 μL each) from two injured and two control animals were run on a 12.5% polyacrylamide gel (Bio-Rad Life Science, Hercules, CA, USA), and transferred overnight onto PVDF. Westerns were run in duplicate and probed with a polyclonal antibody to MMP-8 (Abcam, Cambridge, MA, USA). Blots were also probed for Transferrin (Santa Cruz Biotechnology, Santa Cruz, CA, USA), previously shown to be unaffected by SCI [27], as a loading control. Rat lung lysate was loaded as a positive control for MMP-8.

#### ** *TIMP-1 western blot* **

CSF from injured and control animals (samples not included in multiplex analysis) were in the same way as MMP-8 western blots (above). Lysate from 293 T cells over-expressing TIMP-1 (Santa Cruz) and rat whole brain lysate (Abcam) were used for positive controls. The blot was cut and probed separately for transferrin and TIMP-1 (Santa Cruz Biotechnology).

## **Results**

### **Microarray data**

Out of 96 probe sets tested (48 spots with replicates), seven proteins showed a statistically significant difference in median signal intensity (background subtracted) in at least one technical replicate (the response). Proteins with significant results at the 5% level are reported in Table 2. Responses from both technical replicates are shown for comparison along with the corresponding raw and adjusted *P*-values. IL-1α, Leptin, B7-2/CD86, GM-CSF, IL-1β, MMP-8, and TIMP-1 were all increased in CSF of injured animals compared with controls. Of these results, only IL-1α, MMP-8, and TIMP-1 showed significant increases across both replicate sets after Benjamini-Hochburg adjustment. As expected, there were no statistically significant differences among control datasets (data not shown). Bonferroni adjusted *P*-values are shown for comparison.

**Table 2:** **Simple model: two sample equal variance *t* -tests**

**ID**	**Response Injured**	**Response non-Injured**	**Raw *P* -value**	**BH adj. *P* -value**	**Bonferroni adj. *P* -value**
*IL-1α*	*418.75*	*349.25*	*0.0019*	*0.0287*	*0.3674*
*IL-1α*	*438.75*	*368.00*	*0.0024*	*0.0287*	*1.0000*
Leptin	839.75	733.50	0.0005	0.0156	0.1149
Leptin	838.00	763.00	0.2189	0.3649	1.0000
B7-2/CD86	504.25	386.50	0.0115	0.0927	0.1818
B7-2/CD86	533.75	413.50	0.0038	0.0367	0.2296
GM-CSF	1223.25	1012.00	0.0132	0.0932	0.2194
GM-CSF	1204.75	1002.50	0.0012	0.0287	1.0000
IL-1β	941.50	823.75	0.0200	0.1121	0.0468
IL-1β	992.25	818.00	0.0023	0.0287	1.0000
*MMP-8*	*36424.75*	3360.25	*0.0003*	*0.0123*	*0.0205*
*MMP-8*	*36255.50*	3567.75	*0.0002*	*0.0123*	*0.0246*
*TIMP-1*	*4181.50*	1720.50	*0.0028*	*0.0304*	*0.2153*
*TIMP-1*	*4099.50*	1688.25	*0.0022*	*0.0287*	*0.2732*

Mean response by injury status with raw and adjusted *P*-values for significance between injured and non-injured animals. Response is the difference between the median foreground signal and the median background signal on the Cy3 channel. Raw *P*-values are shown with Benjamini-Hochburg adjustments. Bonferroni adjusted *P*-values are shown for comparison. Proteins in which changes in both technical replicates were significant at the 5% level (BH adjusted *P*-value) are italicized.

### **Microarray data**

**Figure 3:**
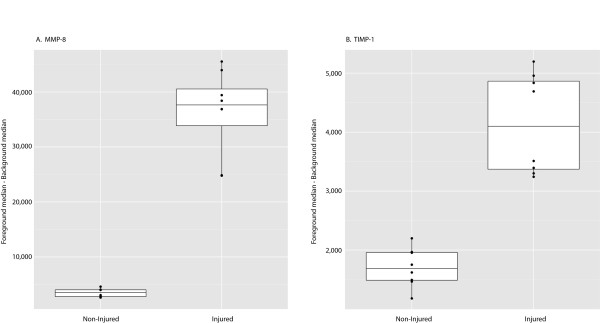
**Array data for MMP-8 (A) and TIMP-1 (B) represented in box plot format.** The plots illustrate the distribution of the median background subtracted signal for the eight measurements (four rats × two reps) in each of the Injured/Control groups. The height of the box plot gives a measure of spread MMP, matrix metalloproteinase; TIMP-1, tissue inhibitor of metalloproteinase-1.

To account for technical replicates, as well as regional signal variability across the array as a whole, a linear mixed model was applied to the data. Table 3 displays the full table of results from the mixed model. The value displayed is the difference between the average of median signal intensity minus the median background intensity (across all technical replicates) for injured and control animals. Positive values indicate an increase in protein in injured animals, and negative values indicate a decrease. Results found to have statistically different signals between the injured and non-injured animals based on raw *P*-values are highlighted. In this model, only MMP-8 and Thymus Chemokine-1 showed statistically significant changes based on raw *P-*values. MMP-8 was elevated in injured animals: the same conclusion as from the probe set level tests (simple model). Thymus Chemokine-1 did not reach significance in the simple model, but showed a statistically significant decrease in the linear mixed model based on the raw *P-*value; a result that did not hold up after Benjamini-Hochburg adjustment. This discrepancy is the result of significant variability within the Thymus Chemokine-1 dataset combined with large differences in the magnitude of signal between injured and control animals. After Benjamini-Hochburg correction of mixed model data, MMP-8 was the only cytokine with a significant change between injured and non-injured animals (*P* < 0.0001). Figure 3 shows all median response data (*n* = 8) from the MMP-8 and TIMP-1 datasets in box plot format.

**Table 3:** Linear mixed model results

**ID**	**Response difference**	**Raw *P-* value**	**BH adjusted *P-* value**
*MMP-8*	*32856.83*	*0.0000*	*0.0000*
Thymus Chemokine-1	−8426.12	0.0163	0.3094
MCP-1	4878.50	0.1624	0.9978
TIMP-1	2416.63	0.4882	0.9978
IL-13	−1482.87	0.6705	0.9978
Agrin	658.13	0.8502	0.9978
TNF-α	383.13	0.9124	0.9978
Fractalkine	214.00	0.9510	0.9978
PDGF-AA	196.13	0.9551	0.9978
GM-CSF	187.25	0.9571	0.9978
IL-4	175.63	0.9598	0.9978
IL-2	171.75	0.9607	0.9978
VEGF	160.25	0.9633	0.9978
ICAM-1	152.88	0.9650	0.9978
Fas ligand	−138.75	0.9682	0.9978
IL-1β	126.50	0.9710	0.9978
LIX	124.75	0.9714	0.9978
β-NGF	120.75	0.9724	0.9978
L-selectin	118.38	0.9729	0.9978
MIP-3α	109.38	0.9750	0.9978
IL-10	100.13	0.9771	0.9978
B7-2/CD86	99.50	0.9772	0.9978
IL-1 R6	97.63	0.9776	0.9978
Leptin	71.13	0.9837	0.9978
IL-6	58.38	0.9866	0.9978
IL-1α	50.63	0.9884	0.9978
RAGE	30.00	0.9931	0.9978
Activin A	19.50	0.9939	0.9978
CINC-2α	−26.37	0.9940	0.9978
Prolactin-R	24.13	0.9945	0.9978
CINC-3	20.88	0.9952	0.9978
CNTF	16.63	0.9962	0.9978
CINC-1	14.63	0.9967	0.9978
IFN-γ	9.63	0.9978	0.9978

BH, Benjamini-Hochburg; MMP, matrix metalloproteinase; TIMP-1, tissue inhibitor of metalloproteinase-1.

Full table of results from mixed model sorted by raw *P*-value. The differences in means between injured and non-injured animals are displayed in the second column. Proteins with changes significant at the 5% level (BH adjusted *P-*values) are italicized.

### **Western blot analysis**

**Figure 4:**
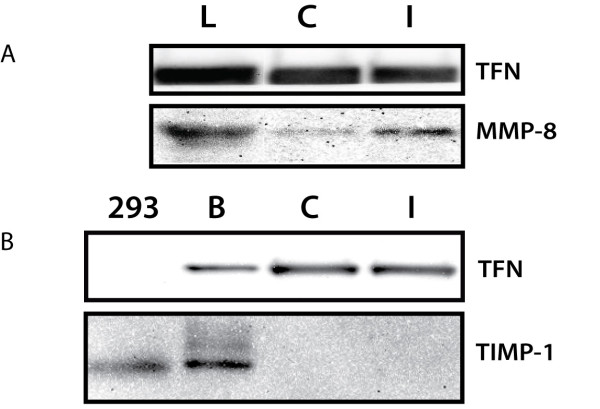
**CSF western blot analysis. (a)** MMP-8 levels are elevated in injured animals (I) compared with controls (C) 12 days post-contusion (*n* = 4 for each condition). Rat lung lysate (L) was loaded as a positive control. Blots were probed for Transferrin as a loading control. **(b)** TIMP-1 is observed in over-expressing 293 cells (293) and rat brain lysate (B), but not in CSF CSF, cerebrospinal fluid; MMP, matrix metalloproteinase; TIMP-1**,** tissue inhibitor of metalloproteinase-1.

To confirm that microarray samples contained a protein with the correct molecular weight for MMP-8, CSF from injured and control animals were run and probed for MMP-8. A probe for Transferrin was used as a loading control. Westerns for both MMP-8 and Transferrin were aligned to create Figure 4(a). One representative set of data is shown. Western blot for MMP-8 shows a band at approximately 53 kD (predicted size for rat MMP-8) in the lung positive control, as well as bands of the same size in each CSF sample. A clear increase in MMP-8 protein was observed in samples from injured animals compared with controls, but more importantly there was no evidence of immune-reactive proteins or breakdown products. The Transferrin blot shows comparable total protein concentrations in each sample as indicated by relative band size/intensity.

Western blot for TIMP-1 was also performed to test for type II errors in the statistical analysis. Specifically we were interested in testing whether there were hits present in the simple model that did not reach significance in the mixed model. The western blot for TIMP-1 along with Transferrin loading controls are shown in Figure 4(b). Bands at approximately 25 kD are seen in positive controls (cells overexpressing TIMP-1 and brain extracts) but not in CSF samples. This result is not unexpected given the relatively weak signal intensity for TIMP-1 in the multiplex array compared with MMP-8, which indicated lower protein concentrations (or weaker antigen-antibody affinity) that were likely below the limit of detection by our western blot system.

## **Discussion**

The primary objective of this study was to characterize the inflammatory profile present in CSF at a subacute time point in a clinically relevant rodent model of traumatic SCI. Our other aim was to test a microarray proteomics platform specifically for this application. Studying inflammation at 12 days post-SCI is highly relevant in that it falls within a clinical ‘window of opportunity’ where patients have been stabilized and are generally more receptive to therapy. We also agree with the SCI field in general that CSF is an important source of potential biomarkers given that it is safe to obtain, readily available, and in close proximity to neuronal tissue of interest. Using small volumes of CSF we were able to study 34 cytokines/growth factors simultaneously, detecting significant changes in seven inflammatory markers. After applying statistical models designed for multiplex testing, we also found robust increases in MMP-8 within CSF, which to our knowledge represents a novel subacute phase biomarker for SCI.

As with any new technology, comparison of results to previous data is crucial in assessing the reliability of the new system. It is generally accepted that a multiphasic inflammatory response occurs after SCI: an early phase peaking at 24 hours and extending from 9 to 10 days, and a later phase between 14 and 180 days post-injury [31]. Studies of inflammation in the setting of SCI have primarily focused their analysis on this early phase between 0 and 72 hours post-injury when neuroprotective strategies are most effective, making comparisons to this study somewhat difficult. Changes in mRNA from rat cord lysate observed by Streit *et al.* (IL-1β, TNF-α, IL-6, and M-CSF) were transient, returning to normal levels by 24 hours post-injury, and remaining at these levels for the remainder of their analysis (10 days) [24]. In this study, TGF-β levels started to rise at 24 hours, peaked at 7days, and remained elevated at 10 days; TGF-β was not included in our analysis. The study by Wang *et al.* showed elevated IL-1β protein in CSF at one hour [25]. Their result differed from Streit *et al.* in that protein levels remained elevated at 7 days, but fits nicely with our IL-1β simple model result. Although changes in IL-1β did not hold up to mixed model analysis, it is quite possible that this result is real, and that we are observing a lull in protein concentration between early- and late-phase inflammation. Further analysis is needed to confirm this result. Yang *et al.* showed elevated levels of IL-1β, IL-6, and TNF-α by western blot from cord lysates, a result that peaked at 6 hours, returned to normal by 24 hours, and was dependent on injury severity [6]. IL-6 and TNF-α levels in our study, although elevated in injured animals, did not reach statistical significance. Finally, the human CSF study by Kwon and colleagues did not show any changes in IL-1β or GM-CSF protein over a 5-day period [26] (both elevated in our simple model at 12 days), a difference that may be explained by the observation window, and/or cross-species differences in the inflammatory response.

MMPs and their endogenous inhibitors (TIMPs) have been studied extensively in the setting of SCI. de Castro *et al.* showed increases in gelatinases MMP-9 and MMP-2 at 12 to14 hours, and 5 days respectively in injured rat spinal cord by zymography. They suggest (using neutralizing antibody studies) that these MMPs are released by infiltrating neutrophils responding to inflammation [32]. Wells *et al.* performed a comprehensive MMP/TIMP mRNA profile in an acute murine model of SCI. They showed an increase in MMP-3, 7, 10, 11, 19, and 20 at 24 hours post-SCI, and elevated levels of MMP-2, 12, and 13, but not MMP-8 at 5 days, colocalizing MMP-12 mRNA to Iba1 positive macrophages in the lesion epicenter [33].Using microarray proteomics, we present a new finding, an increase in MMP-8 protein, also known as neutrophil collagenase or collagenase-2, in CSF at 12 days after SCI in rats. This result is consistent with findings of Veeravalli *et al.* who showed an increase in MMP-8 mRNA following SCI between 3 and 21 days post-injury in mouse cord lysate samples [34]. Increases in TGF- β mRNA 10 days post-SCI reported by Streit *et al.*[24] may in part explain the elevated levels of MMP-8 in our study, as TGF- β is a known inducer of other collagenases like MMP-13, through the Smad3 pathway [35], as well as MMP-2, and MMP-9 though ERK-1/2, and p38 MAPK [36]. In the Streit study [24], TGF-β levels started to rise at 24 hours, peaked at 7 days, and remained elevated at 10 days; TGF-β however was not included in our analysis. Our finding also correlates with data from the stroke field, where Cuadrado and colleagues reported upregulation of MMP-8 protein (and other MMPs) in ischemic brain tissue [37]. Finally, the fact that increases in CSF MMP-8 protein levels were not reported by Lubieniecka *et al.*[27], despite the use of sensitive mass spectrometry analysis at time points ranging from 12 to 72 hours after injury, indicates that our observed increase in MMP-8 protein in CSF represents a novel subacute-phase SCI biomarker. Increases in a variety of MMPs within the spinal cord following SCI are thought to significantly contribute to further tissue damage particularly at acute time points after SCI. At present, however, relatively little is known about CSF levels of specific MMP mRNAs and proteins or their impact on tissue damage at subacute time points after SCI. Elevated levels of MMP-12 mRNA in spinal cord injured tissue observed by Wells and colleagues [33] have been shown to correlate with disruption of the BSCB, however this study did not extend beyond 5 days post-SCI. Whether the significant increase in MMP-8 protein we have observed in the CSF contributes to BSCB disruption and other mechanisms of tissue damage post-SCI requires further investigation beyond the scope of the present study.

Comparing MMP-8 and TIMP-1 signals in both microarray statistical models illustrates the importance of data interpretation in multiplex testing. MMP-8, by far the greatest protein change detected, was significant across both models, suggesting the ability of the array and mixed statistical model combination to correctly identify true changes in the dataset. We would like to reiterate the importance of methods like western blot in verifying protein size when using an ELISA-based system because other immunoreactive proteins may disrupt the analysis.

TIMP-1, by contrast, only reached statistical significance in our simple model. Lack of significance in the linear mixed model indicates that this result is more likely to be a type 1 error than a true positive. Western blot was performed to test this hypothesis. Although it is possible that our inability to detect TIMP by western blot reflects the ability of the mixed model to control the FDR, it is also possible that TIMP-1 is truly elevated following SCI and, as discussed above, the microarray was just more sensitive at identifying biologically significant changes in this molecule.

Clearly, uncertainty exists when interpreting the results of multiplex testing. Different statistical treatment of the data gives us clues, but follow-up testing is often required by conventional methods. This is especially true when using the Benjamini-Hochburg correction, accepting a certain number of false positives per assay. However, there are ways, within the confines of the multiplex format, to reduce uncertainty. One obvious option would be to increase the sample size of each dataset. If we were to run two chips instead of one would we not increase our power to detect differences in the data? This brings up the concept of batch variability: the observation of significant variation between batches (chips in our experiment). This variability commonly offsets any statistical ‘gain’ from the corresponding increase in sample size. A better alternative would be to run sequential arrays, one for screening, as presented here, and another ‘confirmation array’, where only hits from the screening array are spotted, in greater numbers. This sequential approach, although more expensive and time consuming, avoids the problem of batch variation, and prevents controls for the FDR from making costly type II errors.

## **Conclusions**

To our knowledge our study is the first to demonstrate significant increases in MMP-8 protein in CSF after traumatic injury to the spinal cord. We propose that MMP-8 is a novel subacute-phase CSF biomarker for SCI. In addition, our study supports the use of sandwich microarray proteomics as a valid approach for studying CSF in the setting of SCI in rats, and potentially human SCI patients. This assay reliably detects low levels of CSF protein, and has several advantages over conventional methods including quick and easy protocol, high-throughput format, and use of relatively small amounts of precious sample. Disadvantages include relative cost compared with traditional methods, and the need to confirm hits (either with repeat microarray analysis or other assays). Moreover our study stresses the importance of controlling the FDR given the multiplex format. In summary, this assay has the potential to detect new target molecules, characterize complex time-dependent pathophysiologic responses to SCI (like inflammation) and, as more therapies reach the clinic, identify biomarkers with which to assess injury severity and grade response to treatment.

## **Abbreviations**

ASIA, American Spinal Injury Association; BH, Benjamini-Hochburg multiple testing correction; BSCB, blood-spinal cord barrier; CNS, central nervous system; CSF, cerebrospinal fluid; ELISA, enzyme-linked immunosorbent assay; FDR, false discovery rate; IL, interleukin; MMP, matrix metalloproteinase; mRNA, messenger RNA; PCR, polymerase chain reaction; SCI, spinal cord injury; TIMP, tissue inhibitor of metalloproteinase; TNF, tumor necrosis factor.

## **Competing interests**

The authors declare that they have no competing interests.

## **Authors’ contributions**

ML, KM, and SD designed the study. KM conducted all animal injuries and KM and ML performed CSF extractions. ML and KJ performed the microarray and western blot analysis. PD performed all statistical analysis. Manuscript was written by ML, PD, KM and SD. All authors have read and approved the final manuscript.
